# Association of kinase insert domain-containing receptor (*KDR*) gene polymorphism/ haplotypes with recurrent spontaneous abortion and genetic structure 

**Published:** 2015-12

**Authors:** Shiva Shahsavari, Zahra Noormohammadi, Shohreh Zare Karizi

**Affiliations:** 1 *Department of Biology, School of Sciences, Science and Research Branch, Islamic Azad University, Tehran, Iran.*; 2 *Department of Biology, Pishva Branch, Islamic Azad University, Varamin, Iran.*

**Keywords:** *Abortion*, *Genetic structures*, *Polymorphism*, *Restriction fragment length polymorphism*, *VEGF receptor 2*

## Abstract

**Background:**

Recurrent spontaneous abortion is one of the diseases that can lead to physical, psychological, and, economical problems for both individuals and society. Recently a few numbers of genetic polymorphisms in kinase insert domain-containing receptor (*KDR*) gene are examined that can endanger the life of the fetus in pregnant women.

**Objective:**

The risk of *KDR* gene polymorphisms was investigated in Iranian women with idiopathic recurrent spontaneous abortion (RSA).

**Materials and Methods:**

A case controlled study was performed. One hundred idiopathic recurrent spontaneous abortion patients with at least two consecutive pregnancy losses before 20 weeks of gestational age with normal karyotypes were included in the study. Also, 100 healthy women with at least one natural pregnancy were studied as control group. Two functional SNPs located in *KDR* gene; rs1870377 (Q472H), and rs2305948 (V297I) as well as one tag SNP in the intron region (rs6838752) were genotyped by using PCR based restriction fragment length polymorphism (PCR-RFLP) technique. Haplotype frequency was determined for these three SNPs’ genotypes. Analysis of genetic STRUCTURE and K means clustering were performed to study genetic variation.

**Results:**

Functional SNP (rs1870377) was highly linked to tag SNP (rs6838752) (D´ value=0. 214; χ^2^ = 16.44, p<0. 001). K means clustering showed that k = 8 as the best fit for the optimal number of genetic subgroups in our studied materials. This result was in agreement with Neighbor Joining cluster analysis.

**Conclusion:**

In our study, the allele and genotype frequencies were not associated with RSA between patient and control individuals. Inconsistent results in different populations with different allele frequencies among RSA patients and controls may be due to ethnic variation and used sample size.

## Introduction

The Practice Committee of the American Society for Reproductive Medicine (ASRM) has been defined recurrent spontaneous abortion (RSA) to include two or more consecutive pregnancy losses before 20 weeks of gestation (1). The systematic studies have been estimated that 1% of women in reproductive age are affected by RSA. Fetal chromosomal abnormalities, gene mutations, infectious agents and environmental factors such as smoking, alcohol and excessive caffeine consumptions may play important role in recurrent abortions (2). Despite of these records, etiology of RSA (up to 50% of cases) remains undetermined and it is likely to be multifactorial (3, 4).

Placental circulation is pivotal for maintaining pregnancy during implantation and embryo development. Vascular endothelial growth factor (VEGF) as angiogenic factor may associate with RSA in several ethnic groups (5, 6). VEGF plays a main role in fetal and placental angiogenesis, which is secreted from different cells, such as endometrium, placenta as well as endothelial cells and vascular smooth muscle cells (7). Kinase insert domain containing receptor (KDR) is also called VGEF receptor 2 has been reported to be associated with RSA because of angiogenic effects on placenta via the VGEF-KDR pathway (5, 6, 8). Several single nucleotide polymorphisms (SNPs) of the *KDR* gene have been reported in various diseases such as non-small cell lung cancer, breast cancer, coronary heart disease, moyamoya disease and RSA. These SNPs include SNP 32662 C/T in intron region, 1192G/A in exon 7 with the Val297Ile substitution and 1719A/T in exon 11 with the Gln472His substitution, -604T/C at the *KDR* promoter region and -271 G> A polymorphism (8-11). The aim of the present study was to investigate the association between allele frequency of *KDR* polymorphism, including 1192G/A (rs2305948), 32662 C/T (rs6838752) and 1719A/T (rs1870377) in RSA Iranian women. Genetic structure based on *KDR* polymorphisms in the population was studied as well.

## Materials and methods

This case control study was carried out in Tehran Medical Genetic Center, Tehran, Iran between 2010-2011. Women aged 17-45 years old who were diagnosed as idiopathic recurrent spontaneous abortion (RSA) (at least two consecutive pregnancy losses before 20 weeks of gestational age) according to the ASRM definition (4) and no male factor cause were included. Patients who suffered from a chromosomal abnormality like translocation, trisomy, and triploidy were excluded group and finally 100 participants were enrolled as case group. A hundred women with the same age range (17-45 years), having regular menstrual cycles, at least one naturally natural pregnancy, and normal karyotype were chosen as control group. This study was approved by Tehran Medical Genetic Center Ethics Committee. All patients gave written informed consent for participation in this study.

**Analysis of** ***KDR*** **polymorphism**

Genomic DNA was extracted from 5 ml Ethylenediaminetetraacetic acid (EDTA) anti-coagulated peripheral blood using the standard salting-out procedure. The quantity and quality of extracted DNA were examined by Nanodrop spectrophotometer and 0.8% agarose gel electrophoresis, respectively.

Two functional SNPs were located in *KDR* gene; rs1870377 (Q472H), and rs2305948 (V297I) as well as one tag SNP in the intron region (rs6838752) were genotyped using a PCR based restriction fragment length polymorphism (PCR-RFLP) analysis. Oligonucleotide primers were designed based on the published sequence of human *KDR* gene (NCBI Reference Sequence: NG_012004.1) by using Gene Runner ver. 3.05 software (Table I).

**Figure 1:**
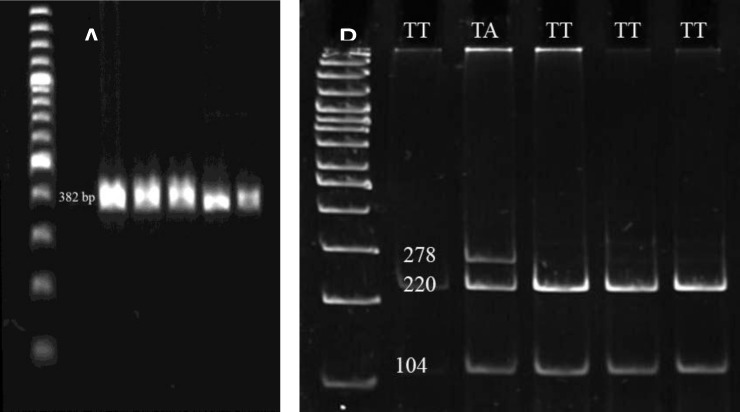
PCR products of SNP rs1870377 region (382 bp) on 1.2% agaros gel (A) and, restriction fragments produced by AluI digestion (B) on 10% PAGE. The 100 bp DNA ladder was used. Bands less than 60 bps are not visualized on gel electrophoresis.

**Figure 2:**
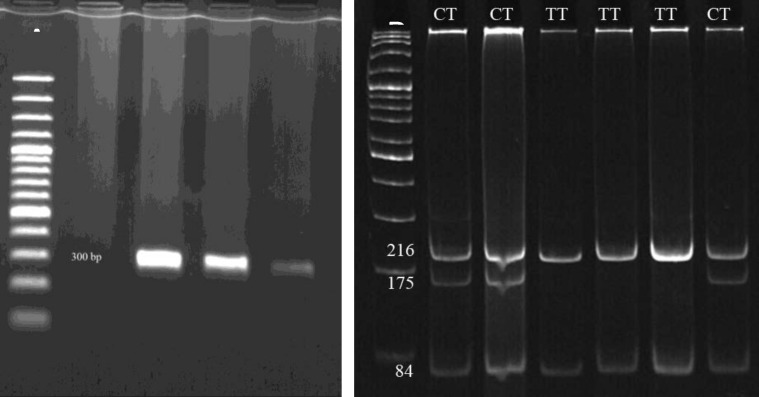
PCR products of SNP rs6838752 region (300bp) on 1.2% agaros gel (A) and, restriction fragments produced by AluI digestion (B) on 10% PAGE. The 100 bp DNA ladder was used. Bands less than 60 bps are not visualized on gel electrophoresis

**Figure 3:**
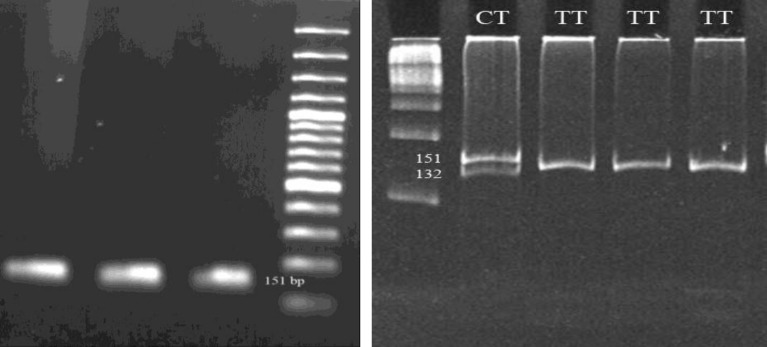
PCR products of SNP rs2305948 region (151 bp) on 1.2% agaros gel (A) and, restriction fragments produced by AluI digestion (B) on 10% PAGE. The 100 bp DNA ladder was used. Bands less than 60 bps are not visualized on gel electrophoresis

The PCR was carried out in a total 25 µl volume containing 50 ng of the genomic DNA, 1x PCR buffer, 0.23 mM dNTP-Mix, 6 pmol of each primer, 1.3 mM MgCl2, and 1 U/µl Taq DNA polymerase. The thermal cycler program was performed for 5 min at 95^°^C as initiation denaturation following 30 cycles of 1 min at 95^°^C, 1 min at annealing temperature based on primer pairs (Table I, Figures 1A, 2A, and 3A) and 1 min in 72^°^C with a final extension at 72^°^C for 10 min. 

Restriction map for each PCR reaction was constructed using the New England Biolabs cutter program. AluI, MnlI, and RsaI restriction endonucleases were chosen for genotype analysis of rs1870377, rs6838752 and rs2305948, respectively. Digestion reaction was carried out according to manufacture procedure (Fermentas) and incubated at 37^°^C for 16h. Restriction digestion band sizes have been provided in table I and figures 1B, 2B and 3B.

Allele profiles were visualized by running on 10% poly acrylamide gel electrophoresis (PAGE) following staining with Syber Green gel stain. 100bp DNA Ladder Plus (Fermentas) was used as a size marker.

**Statistical analysis**

Differences in three SNPs’ frequencies were evaluated between the control and case samples by using χ2 or Fisher’s exact test. It was used crude odds ratios (COR) and 95% confidence intervals (CI) as a calculating the association between genotypes and recurrent spontaneous abortion. The p-value< 0.05 was considered to be statistically significant. Other inheritance models like Akaike’s Information Criterion (AIC) and Bayesian Information Criterion (BIC) were calculated for the selection of the best model for a specific SNP. Hardy–Weinberg equilibrium was estimated with standard procedure using Chi square test.

Haplotype frequency for three SNPs’ genotypes as well as haplotype association between the control and case samples were estimated using SNPstats software (12). In the present study, for linkage disequilibrium (LD) of three SNPs studied, selected statistics (D´, Pearson’s r and associated p-value) between each pair of SNPs were analyzed by SNPAlyze 7.0. Pro software (DYNACOM Co., Ltd., Yokohama, Japan).

Alleles of each genotype were treated as binary characters and coded accordingly (presence = 1, absence = 0). Dice as well as the Nei’s genetic distance (13, 14), were determined among the studied case and control samples. These distances were used for the grouping of individuals by an unweighted paired group method with arithmetic average (UPGMA) and Neighbor Joining clustering methods after 100 times of bootstrapping (14).

The AMOVA (Analysis of Molecular Variance) test was performed to show the genetic differences among the studied groups (with 1000 permutations) as performed in GenAlex ver. 6.4 (15).

The STRUCTURE Harvester web site (16) was used to visualize the STRUCTURE results and also to perform Evanno method to identify a proper number of K (17).

Two summary statistics were used to present K-Means clustering, 1- pseudo-F (18) and 2- Bayesian Information Criterion (19). Pseudo-F relates r2 is the fraction of the total variance that is explained by the clustering. The clustering with the highest value for pseudo-F is regarded to provide the best fit (20).

## Results

This study was performed on 100 women with 17-45 years and mean age ±SD, 28 ±5.22 years who were diagnosed as RSA. The mean ± SD of abortion number was 2.2±0.99 (range 2-6). One hundred women at the same age range with all normal criteria which mentioned in materials and methods section was used as control group.

Allele and genotype distributions of three KDR SNPs are listed in table II. The ancestral alleles in all 3 SNPs were the highest allele frequencies in both case and control samples while A, C, and C alleles were minor allele frequencies (MAF) with 15%, 16% and 34%, respectively (Table II). Chi square test showed no significant differences between case and control allele frequencies in rs6838752 and rs2305948 *KDR* gene SNPs (p=0.43 and 0.35 respectively, Table II) while, rs1870377 with χ2=3.249, showed a slightly significant difference (p=0.08).

The estimated risk of subjects with one or two copies of risk alleles in different inheritance models (dominant for rs1870377 and rs6838752; dominant, co-dominant, over-dominant and recessive for rs2305948 *KDR* SNP) showed no significant differences for crude odds ratios in 95% confidence interval (OR=1.62, 1.05 for rs1870377 and rs6838752, respectively and OR=1.04, 0.89 and 0.76 for rs2305948 in three models of inheritance: dominant, recessive and overdominant respectively, Table III). AIC and BIC were also calculated for selection the best inheritance model in rs2305948.

LD based on D´ value, r and Chi square, associated p-value and AIC were analyzed between SNP pairs (Table IV). Functional SNP (rs1870377) showed highly linked to tag SNP (rs6838752) (D´ value=0.214; χ2=16.44, p< 0.001) while no linkage was estimated in other SNP pairs.

Eight different haplotypes of three (rs1870377, rs6838752 and rs2305948) loci of *KDR* gene were observed in both case and control subjects. The T-T-T haplotype (ancestral alleles in each site) was more frequent in both case and control samples (49%, Table V). The estimated risk in 95% CI for all 8 haplotypes was not shown a significant association in RSA patients. The lowest frequent haplotype in the patient group was A-C-T with 2% frequency while in the control group, A-C-C haplotype (consists of MAF in each site) showed the lowest frequent haplotype (1%).

The study of genetic variation based on three nucleotide sites in *KDR* gene resulted high genetic polymorphism (77%) in both sample groups (case and control). Analysis of molecular variance (AMOVA) was performed to estimate genetic differentiation between and among two groups. In total, 100% of total variance was due to within group variation and no significant differences (p=0.478) were observed among the groups studied (Table VI).

**Figure 4:**
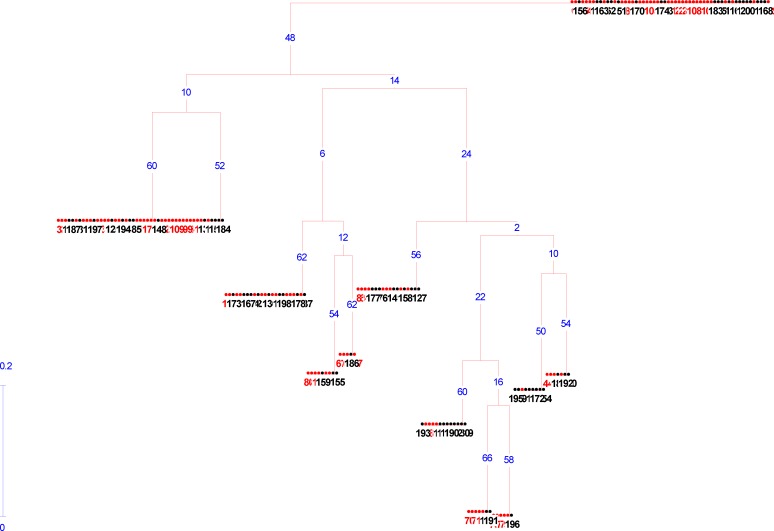
Neighbor Joining analysis based on three SNPs studied for case and control samples. 1 to 100= patient individuals; 101 to 200= control individuals. bootstrapping numbers on branches

Grouping of genotypes using the Neighbor Joining clustering method is depicted in figure 4. In general 4 distant groups were formed. In all clusters, there is not distinct separation between case and control samples, which is in agreement with the result of AMOVA. In detail, the individuals in each cluster grouped into two or four sub-clusters showing genetic variations among the samples.

**Figure 5:**
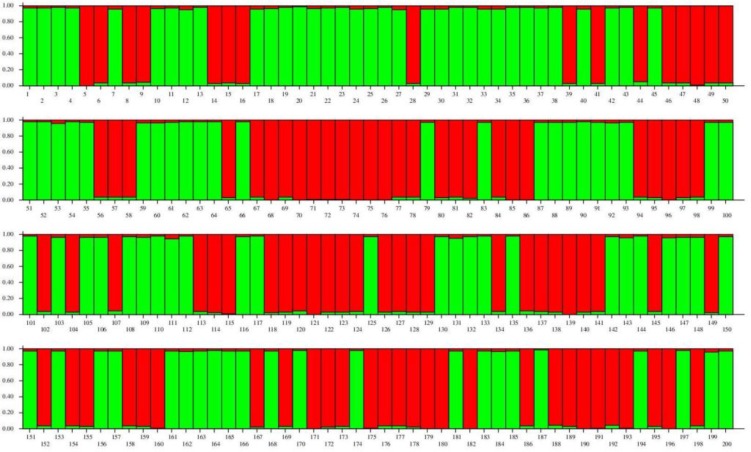
STRUCTURE plot based on three KDR SNP sites. Different alleles show in different colors. 1 to 100= patient individuals; 101 to 200= control individuals

STRUCTURE result and Evano test also partitioned the studied case and control samples in 3 genetically distinct clusters. The STRUCTURE plot of 200 individuals showed the presence of 2 kinds of allelic composition in two groups (different color in figure 5). This result indicated that the case and control samples gained similar combinations of alleles. 

The result of K-Means clustering is presented in Table VII. It showed the highest value of pseudo-F (232.64) for K=8, and the lowest value of BIC (726.157) for K=8. Therefore, it showed K=8 as the best fit for the optimal number of genetic subgroups in our studied materials. 

**Table I:** Primer sequences designed for three SNPs of *KDR* gene with their PCR length, annealing temperature (Ta), restriction enzymes and, restriction digestion band size for each genotype. AluI and MnlI enzymes digested two sites of PCR products. (Underlined numbers show restriction bands belong to rs1870377 and rs6838752 sites

**KDR gene (SNP)**	**Primer sequence**	**PCR length (bp)**	**Ta**	**Restriction enzyme**	**Restriction digestion** **band size (bp)**
1719A→T (rs1870377,exon 7)	F-TGCAAGTCCTCCACACTTCTCCAT R-AAGGAGGCCAGTGGCTTCTAAGTT	382	63	AluI	AA:104, 278 AT: 58, 104, 220, 278 TT: 58,104, 220
32662 C→T (rs6838752, intron)	F- GAGTCAACAACAACAGCAACAAG R- TGACAAATGTGGTGTATTCAGATG	300	60	MnlI	CC: 41, 84,175 CT: 41, 84,175, 216 TT: 84, 216
1192C→T (rs2305948, exon 11)	F-ATCCTTGGTCACTCCGGGGTA R- TATGCTGTGCTTTGGAAGTTCAG	151	58	RsaI	CC: 19, 132 CT: 19, 132, 151 TT: 151

PCR: polymerase chain reaction

**Table II:** Allele and genotype frequencies in the case and control samples in three SNPs studied

**SNP**	**All subjects**		**Case group**		**Control group**		
**Allele/Genotype**	**Count**	**Proportion**	**Count**	**Proportion**	**Count**	**Proportion**	**Chi square (p-value)** *
**rs1870377**							
T	340	0.85	175	0.88	165	0.82	3.249 (0.08)
A**	60	0.15	25	0.12	35	0.18	
TA	60	0.3	25	0.25	35	0.35	
TT	140	0.7	75	0.75	65	0.65	
**rs6838752**							
T	337	0.84	169	0.84	168	0.84	0.230 (0.43)
C^**^	63	0.16	31	0.16	32	0.16	
TC	63	0.32	31	0.31	32	0.32	
TT	137	0.68	69	0.69	68	0.68	
**rs2305948**							
T	262	0.66	127	0.64	135	0.68	0.705 (0.35)
C**	138	0.34	73	0.36	65	0.32	
CC	47	0.24	26	0.26	21	0.21	
TC	44	0.22	21	0.21	23	0.23	
TT	109	0.55	53	0.53	56	0.56	

* Chi square (X^2^) calculated based on 2× 2 Contingency Table using SNPstats software.

** Minimum allele frequency (MAF allele).

**Table III:** Inheritance models of SNPs and odds ratio (OR) with 95% confidential interval between case and controls

**Model**	**Genotype**	**Case group** **N (%)**	**Control group** **n (%)**	**OR (95% CI)**	**p-value**	**AIC**	**BIC**
**rs1870377**							
**---**	TT	75 (75%)	65 (65%)	1	0.12	278.9	285.5
	AT	25 (25%)	35 (35%)	1.62 (0.88-2.98)			
**rs6838752**							
**---**	TT	69 (69%)	68 (68%)	1	0.88	281.2	287.8
	CT	31 (31%)	32 (32%)	1.05 (0.58-1.90)			
**rs2305948**							
Codominant	TT	53 (53%)	56 (56%)	1	0.7	282.6	292.4
	CT	21 (21%)	23 (23%)	1.04 (0.51-2.09)			
	CC	26 (26%)	21 (21%)	0.76 (0.38-1.52)			
Dominant	TT	53 (53%)	56 (56%)	1	0.67	281.1	287.7
	CT-CC	47 (47%)	44 (44%)	0.89 (0.51-1.55)			
Recessive	TT-CT	74 (74%)	79 (79%)	1	0.4	280.6	287.2
	CC	26 (26%)	21 (21%)	0.76 (0.39-1.46)			
Overdominant	TT-CC	79 (79%)	77 (77%)	1	0.73	281.1	287.7
	CT	21 (21%)	23 (23%)	1.12 (0.58-2.20)			
Log-additive	---	---	---	0.89 (0.64-1.24)	0.49	280.8	287.4

Odds ratio (OR) and 95% confidence interval (CI) as a calculating the association between genotypes and recurrent spontaneous abortion using logistic regression analysis. Akaike’s Information Criterion (AIC) and Bayesian Information Criterion (BIC) were calculated for the selection of the best model for a specific SNP.

**Table IV:** linkage disequilibrium analysis between pair SNPs studied

**Pair** **A**	**Pair** **B**	**Allele count**	**D-value**	**D´-value**	**CI 95%**	**r-square**	**Chi-square**	**df**	**p-value**	**AIC**
rs1870378	rs6838752	400	0.027	0.214	0.086-0.343	0.043	16.446	1	0.000	11.876
rs1870378	rs2305948	400	0.000	0.050	-0.122-0.222	0.000	0.459	1	0.498	-0.547
rs6838752	rs2305948	400	0.000	-0.151	-0.452-0.149	0.002	1.163	1	0.280	-0.807

linkage disequilibrium (LD) of SNPs was calculated based on D, D´, Pearson’s r and Chi square values calculated based on 2 x 2 Contingency Table between each pair of SNPs by SNPAlyze 7.0. Pro software. Akaike’s Information Criterion (AIC) is inheritance model was calculated for the selection of the best model for a specific SNP

**Table V:** Haplotype patterns with their frequencies in case and control groups

**Haplotype **	**Total**	**Case group**	**Control group**	**Cumulative frequency**	**OR (95% CI)**	**p-value**
T-T-T	0.4767	0.4909	0.4658	0.4767	1	---
T-T-C	0.2664	0.2852	0.2425	0.7431	0.97 (0.62 - 1.52)	0.89
T-C-T	0.0841	0.0771	0.0917	0.8272	1.32 (0.53 - 3.28)	0.55
A-T-T	0.0664	0.0469	0.0865	0.8936	2.17 (0.76 - 6.16)	0.15
A-T-C	0.0331	0.022	0.0452	0.9266	2.42 (0.58 - 10.03)	0.23
A-C-T	0.0278	0.0202	0.0309	0.9544	1.90 (0.34 - 10.55)	0.47
A-C-C	0.0228	0.036	0.0124	0.9772	0.44 (0.08 - 2.59)	0.37
T-C-C	0.0228	0.0218	0.025	1	1.21 (0.22 - 6.68)	0.83

Odds ratios (OR) with 95% confident interval calculated by logistic regression.

Global haplotype association p-value: 0.49.

**Table VI:** Analysis of molecular variance (AMOVA) test between case and control groups based on three SNP data

**Source**	**Degree of freedom**	**Sum of square**	**Mean of square**	**Estimated variation**	**Percentage of variation**
Among group	1	1.200	1.200	0.000	0
Within group	198	288.320	1.456	1.456	100
Total	199	289.520		1.456	100
Stat	Value	P(rand >= data)			
PhiPT	-0.002	0.487			

AMOVA test used to estimate population differentiation directly from molecular data and testing hypotheses about such differentiation based on squared Euclidean distance

**Table VII:** Clustering statistics from k=1 to k=8 based on SNPs data

**k**	**SSD(T)**	**SSD(AC)**	**SSD(WC)**	**r-squared**	**pseudo-F**	**AIC**	**BIC**
1	289.52	0	0	0	0	76.002	1138.943
2	289.52	88.92	201	0.307	87.767	4.66	1070.859
3	289.52	141.8	148	0.49	94.568	-54.493	1014.943
4	289.52	180.6	109	0.624	108.35	-113.355	959.297
5	289.52	210.2	79.4	0.726	129.11	-174.571	901.275
6	289.52	232.8	56.7	0.804	159.27	-239.618	839.4
7	289.52	246.2	43.3	0.85	182.88	-291.42	790.748
8&*	289.52	259	30.5	0.895	232.64	-359.139	726.157

SSD(T)= sum of square distance (total)

SSD_AP/WG_= sum of square distance (among-populations-within-groups)

Pseudo-F= it relates r-squared, the fraction of total variance that is explained by the clustering, to the number of clusters k and the number of populations

* Best clustering according to Calinski & Harabasz' pseudo-F: k = 8; & Best clustering according to Bayesian Information Criterion: k = 8 Akaike’s Information Criterion (AIC) and Bayesian Information Criterion (BIC)

## Discussion

In the present study, the association between the *KDR* gene and the occurrence of idiopathic recurrent spontaneous abortion were studied using three tag SNPs. Based on dbSNP, the global minor allele frequency (MAF, the second most frequent allele value) of three SNPs studied consists of allele A=0.2346 for rs1870377, allele C=0.2378 for rs6838752 and allele T=0. 1313 for rs2305948 by Hap Map database. The data showed that minor allele frequency of two SNPs (rs1870377, rs6838752) possessed lower frequency than the global MAF, while the third SNP (rs2305948) revealed a higher frequency (Table I). Rah *et al* (8) reported higher frequency of MAF in three hundred twenty-seven idiopathic RSA patients and 230 controls with Korean ethnicity. Based on literatures, minor allele frequency of studied SNPs varied in different populations, which may stem from gene pools studied (5, 8, 11). 

In the present study, the frequency of alleles in three SNPs did not show significant differences between the case and control samples (p> 0.05). In addition, none of genotypes showed susceptibility to recurrent spontaneous abortion. AMOVA test also supported the lack of differentiation between the case and control groups based on allele frequencies. Rah *et al* (8) also reported no association between RSA and *KDR* 1192G/A (rs1870377) or 1719A/T (rs2305948) while the association of 1719A/T with RSA was found in Taiwanese Han women with other Asian ethnicity (5). Two *KDR* functional SNP (SNP1192 and SNP1719) located in exon 7 and exon 11 are the key elements of the *KDR* binding domain of VEGF. Wang *et al* (11) believed that down-regulation of the VEGF/KDR signaling pathway by *KDR* mutations in functional SNP sites may increase the risk of coronary heart disease.

In detail, linkage disequilibrium analysis in SNP pairs revealed that functional SNP (rs1870377, Q472H) showed highly linked to tag SNP (rs6838752). Also, in the Chinese Han population LD block of these two loci was very highly linked (5).

Inconsistent results between two Asian ethnic groups, including Korean and Taiwanese groups (5, 8) as well as the present study (Iranian) with different allele frequencies among RSA patients and controls may be involved two main factors including, ethnic variation and sample size in these studies.

In order to evaluate the genetic variations among samples studied, cluster analysis was performed. The presence of both patients and control samples in all clusters support lack of genetic differentiation among them in three SNPs studied. In more details, sub-grouping of samples is based on eight haplotypes obtained. The K means clustering also estimated eight groups as the best fit of genetic variations.

## Conclusion

In conclusion, our findings did not show *KDR* polymorphism associated with recurrent spontaneous abortion in Iranian patients studied. Although it is believed that *KDR* and its regulation of angiogenesis by the VEGF/KDR pathway may be involved in abortion risk (21-23), It could not supported association of these polymorphisms and abortion in Iranian ethnic population. However, the relationship between *KDR* polymorphism in spontaneous abortion events and *KDR* gene expression as well as studying more *KDR* SNP sites should be considered in further studies.

## Note

***This article extracted from M.Sc. thesis. (Shiva Shahsavari)***
